# A parent focused child obesity prevention intervention improves some mother obesity risk behaviors: the Melbourne inFANT Program

**DOI:** 10.1186/1479-5868-9-100

**Published:** 2012-08-28

**Authors:** Sandrine Lioret, Karen J Campbell, David Crawford, Alison C Spence, Kylie Hesketh, Sarah A McNaughton

**Affiliations:** 1 Centre for Physical Activity and Nutrition Research; C-PAN, School of Exercise and Nutrition Sciences; Deakin University, 221 Burwood Hwy, Burwood, Victoria, 3125, Australia

**Keywords:** Dietary pattern, Physical activity, TV viewing, Randomized controlled trial, Mothers

## Abstract

**Background:**

The diets, physical activity and sedentary behavior levels of both children and adults in Australia are suboptimal. The family environment, as the first ecological niche of children, exerts an important influence on the onset of children’s habits. Parent modeling is one part of this environment and a logical focus for child obesity prevention initiatives. The focus on parent’s own behaviors provides a potential opportunity to decrease obesity risk behaviors in parents as well.

**Objective:**

To assess the effect of a parent-focused early childhood obesity prevention intervention on first-time mothers’ diets, physical activity and TV viewing time.

**Methods:**

The Melbourne InFANT Program is a cluster-randomized controlled trial which involved 542 mothers over their newborn’s first 18 months of life. The intervention focused on parenting skills and strategies, including parental modeling, and aimed to promote development of healthy child and parent behaviors from birth, including healthy diet, increased physical activity and reduced TV viewing time. Data regarding mothers’ diet (food frequency questionnaire), physical activity and TV viewing times (self-reported questionnaire) were collected using validated tools at both baseline and post-intervention. Four dietary patterns were derived at baseline using principal components analyses including frequencies of 55 food groups. Analysis of covariance was used to measure the impact of the intervention.

**Results:**

The scores of both the "High-energy snack and processed foods" and the "High-fat foods" dietary patterns decreased more in the intervention group: -0.22 (−0.42;-0.02) and −0.25 (−0.50;-0.01), respectively. No other significant intervention *vs.* control effects were observed regarding total physical activity, TV viewing time, and the two other dietary patterns, i.e. “Fruits and vegetables” and “Cereals and sweet foods”.

**Conclusions:**

These findings suggest that supporting first-time mothers to promote healthy lifestyle behaviors in their infants impacts maternal dietary intakes positively. Further research needs to assess ways in which we might further enhance those lifestyle behaviors not impacted by the InFANT intervention.

## Background

Prevalence of childhood overweight is of concern worldwide, with rates in school-age children as high as 30% in the Americas and 20% in Europe [1]. In Australia, 25% of school-aged children [2] and 20% of preschool children [3] are overweight, suggesting that the behavioral risk factors that impact energy balance may be important from very early ages. Overweight and associated behavioral determinants also track throughout childhood [4-6].

Children from families where obesity promoting behaviors are prevalent are at higher risk of overweight and obesity [7]. Beyond the shared genetic predisposition [8], children’s behaviors are influenced by parental knowledge, attitudes, and modeling [9-12]. The family environment is the first ecological niche of children and, as such, is likely to exert an important influence on the onset of children’s habits and modeling of their behaviors [13]. In particular, it has been shown that as early as ages six and twelve months, child and maternal diets are associated [14,15]. The importance of these early years suggests it is timely to design obesity prevention interventions that focus on infancy and early childhood, involving parents, and targeting the modifiable behavioral determinants of weight gain in both children and parents. Indeed, targeting infants within their families is likely to be an opportunity to prevent obesity risks in parents as well.

A recent review by Hesketh and Campbell [16] highlights that interventions targeting children under five years of age are scarce, and those including infants even rarer. Those authors note that despite inconsistent results, parents are receptive to positive messages aimed at improving diet, physical activity and sedentary behaviors in their children. In Victoria, Australia, parents make on average 35 visits to health care providers over the first year of their infant’s life [17]. This high level of engagement with the health care system provides a promising frame in which to organize interventions. It is in this context that The Melbourne Infant Feeding Activity and Nutrition Trial (InFANT) Program was developed. This cluster-randomized controlled trial (RCT) involved first-time parents within their existing social networks [18] and used an anticipatory guidance framework [19] which focused on parenting skills and strategies aimed at promoting the development of healthy behaviors (including parental modeling of these behaviors) from early infancy. The current analysis tested the hypotheses that mothers receiving this early childhood obesity prevention intervention would also improve their own dietary patterns and engagement in physical activity and, conversely, decrease their TV viewing behaviors.

## Methods

### Study design

The Melbourne InFANT Program is a cluster-RCT undertaken within the pre-existing first-time parents groups organized by Maternal and Child Health Nurses in Victoria, Australia [18]. A two-stage random sampling design was used to select first-time parents groups across all socio-economic position areas. Fourteen local government areas within a 60 km radius of the research centre (Deakin University in Burwood, Victoria, Australia) were the first units of randomization. Within the local government areas, 62 first-time parents groups were then randomly selected, with probability of selection proportional to the total number of first-time parents groups being offered in that local government area. When first-time parents groups declined to participate, another randomly selected group was approached. Groups that consented to participate were then randomly allocated to intervention or control arms. Randomization was undertaken using a computer generated random number schedule developed by a statistician who had no contact with the centers.

Inclusion criteria were literacy in English and a minimum of eight parents (in fact, mothers) in the groups consenting to participate (six in low Socio-Economic Indices For Areas). Infants with chronic health problems likely to influence height, weight, physical activity or eating behaviors were excluded from the analyses but could participate in the study. Informed written consent was obtained from participating mothers.

InFANT was approved by the Deakin University Human Research Ethics Committee and the Victorian Government Department of Human Services, Office for Children, Research Coordinating Committee.

### Intervention

The intervention, described in detail elsewhere [18], focused on parenting skills and behaviors that aimed to promote the development of healthy eating and physical activity behaviors in infants, along with reduced sedentary behaviors. This dietician-delivered intervention comprised six 2-hour sessions delivered quarterly during the regular meeting time of the first-time parents’ group. Based on the theory of anticipatory guidance [19], the intervention incorporated a range of modes of delivery and educational strategies including brief didactic sessions, use of group discussion and peer support, exploration of perceived barriers and facilitators, use of visual and written messages, and mail-outs. Intervention materials incorporated six purpose designed key messages within a DVD and written handouts: “Eat together, play together”, “Colour every meal with fruit and veg”, “Parents provide, kids decide”, “Tap on water”, “Snack on fruit and veg”, “Off and running”. A newsletter reinforcing key messages was sent to participants between sessions. A range of cognitive feedback activities were employed to promote parental examination of personal eating, physical activity and sedentary behaviors. Emphasis on these behaviors focused on the importance of personal health and on the ways in which parental behaviors would impact subsequent child health behaviors (via parental modeling in this instance).

Control group families received usual care, and newsletters regarding generic issues in child health were sent to participating families three monthly. Researchers met with the control group participants three times over the course of the study to collect data.

### Objectives and outcome measures

The efficacy of the intervention was tested by comparing families allocated to the intervention to those in the control group following the conclusion of the 15 month intervention (when infants were 18 months of age). The outcomes addressed by the current study are the adoption of personal lifestyle behaviors by first-time mothers, specifically healthy eating, increased physical activity, and reduced TV viewing time.

### Measurements

Parental data relating to their own diet, physical activity and TV viewing behaviors were collected using self-administered questionnaires provided to mothers at baseline and post-intervention. Questionnaires were returned and checked by research staff. If mothers did not attend, questionnaires were sent and then returned by mail.

Demographic and socio-economic variables included mothers’ age; marital status; country of birth; main language spoken at home; employment status and education level. Education level was defined in three categories: low (secondary school or below), intermediate (trade and certificate qualifications) or high (university degree or higher). Duration of pregnancy, breastfeeding status at baseline, and mother’s pre-pregnancy weight and height were also self-reported. Maternal pre-pregnancy body mass index (BMI) was calculated as weight/height^2^ (kg/m^2^).

#### Assessment of diet of mothers

Dietary data was collected from mothers at baseline and post-intervention using a food frequency questionnaire (FFQ). This has previously been validated using 7-d food diaries [20]: correlation coefficients for energy-adjusted nutrient intakes ranged from 0.28 (vitamin A) to 0.78 (carbohydrate). This tool is an updated version of the semi-quantitative FFQ specifically developed for the Melbourne Collaborative Cohort Study [21]. Mothers were asked to indicate how often they had consumed each food or beverage item over the preceding 12 months. The FFQ has 10 response options for 98 food items ranging from “never” to “three or more times per day”. These data were converted into daily equivalent frequencies according the Cancer Council Victoria protocol. The FFQ also included 11 additional questions relating to the type and amount of milk consumed (number of glasses per day); the amount of diet and non-diet soft drinks consumed (number of glasses per day); the type and amount of bread consumed (number of slices per day); the number of eggs per week; and the frequency of consumption per week of both alcoholic and hot beverages.

The statistical methods used to assess the overall diet include *a posteriori* approaches such as factor and cluster analyses, and the *a priori* dietary index approach [22]. These methods, which allow multiple components to be assessed simultaneously, have been developed in nutritional epidemiology to overcome the inability of the traditional single-nutrient or single-food approaches to account for the complexity of the diet. Principal component analysis (PCA) has therefore often been used to identify and assess overall food patterns of a given population. This multivariate method was utilized in the current study because - contrary to cluster analysis - this provides continuous variables more suitable for longitudinal analyses.

#### Assessment of mothers’ physical activity

Mothers’ total physical activity was assessed at baseline and post-intervention using a valid [23] and reliable [24] questionnaire. Mothers were asked to estimate the total duration they spent walking continuously (for at least 10 minutes); and doing both vigorous and moderate physical activity the week preceding the interview. According to established survey protocols [25], we calculated total physical activity time (in min/week) by summing the time spent in walking and moderate activity and twice the time spent in vigorous activity. To avoid errors due to over-reporting, any times greater than 840 min/week for a single activity type were truncated at 840 min/week (14 h). In addition, total times in all activities that were greater than 1680 min/week (28 h) were truncated at 1680 min/week.

#### Assessment of mothers’ sedentary behavior

In the self-administered questionnaire, mothers also reported the usual time spent watching television or videos/DVD’s on both weekdays and weekend days. An average daily time (in min/d) was calculated and weighted from the values reported for each type of day. In this article, “watching television or videos/DVD’s” is thus used as a proxy of sedentary behavior. Reported durations and total TV viewing time were truncated at 1080 min/day (18 h).

### Statistical analyses

Based on the assessment of the similarities in food type, energy density and context of consumption, all foods and beverages were assembled into 55 groups (Table1) and frequencies of consumption of foods within each group were summed. Dietary data were standardized by subtracting the mean and dividing by the standard deviation within each of these food groups. Four dietary patterns were derived in first-time mothers at baseline in a previous study [26], accounting for 24% of the explained variance. PCA with varimax rotation included the 55 food groups [27]. The number of components was selected considering eigenvalues >1.0, the scree plot and the interpretability of the patterns [28]. To both interpret the results and calculate the scores, we retained the items most strongly related to each pattern, i.e. those for which the absolute value of the loading coefficient was >0.15. This threshold was chosen accounting for the overall range of loadings observed in our data (i.e. the ranking of foods in the pattern) and both the interpretability and differentiation of each pattern. Dietary pattern labels were allocated according to these most significant items associated with the dietary pattern (Table2). The first pattern was positively correlated with the consumption of vegetables, legumes, non-fried fish, and fruits. This pattern was labeled “Fruits and vegetables”. Pattern two, labeled “High-energy snack and processed foods”, was mainly characterized by high consumption of processed foods, such as pizzas, savory pastries, crisps, Ketchup, etc. The third component had high positive loadings for potatoes cooked with added fat, fat added to vegetables, white bread, fried fish, fat spreads, and full-cream milk. This pattern was named “High-fat foods”. The fourth pattern was a mixed pattern with high loadings for cereals (wholemeal crackers; breakfast cereals), reduced-fat milk, and sweets (ice cream; confectionary other than chocolate-based). This pattern was labeled “Cereals and sweet foods”. Due to the longitudinal design of our analyses, the factor scores for each pattern identified at baseline were calculated at the individual level at both baseline and post-intervention by summing the observed standardized frequencies of consumption per food group at each point in time, weighted according to the factor loadings estimated at baseline. 

**Table 1:** Food classification

**Food groups**	**Items included**
Full cream milk	
Reduced fat milk	
Other milks	Soy milk or others.
Diet carbonated soft drink	
Non-diet carbonated soft drink	
Fruit juices	
White bread	Including high fiber white.
Non white bread	Including wholemeal, multi-grain, rye, soy and linseed.
Wholemeal crackers	
Porridge	
Breakfast cereals	
Rice	
Pasta	
Non-fat Potatoes	
Raw vegetables	
Cooked vegetables	
Legumes	
Common fresh fruits	Bananas, apples, pears, and oranges.
Other fresh fruits	
Tinned or dried fruits	
Ricotta and cottage cheese	
All other cheeses	
Yogurt	
Eggs	
Chicken	
Red meats	Beef or veal (not corned), lamb, and pork.
Sausages	
Deli meats	Processed meats (e.g. ham, corned beef, prosciutto, salami) and bacon.
Non-fried fish	
Fried fish	
Potatoes cooked in fat	
Savory pastries	
Pizzas	
Salty and non-whole meal biscuits	
Crisps	Corn chips, potato crisps, Twisties.
Sweet biscuits	
Cakes and pastries	
Ice cream	
Chocolate-based products	Chocolate and confectionary containing chocolate.
Other confectionery	
Olives	
Peanut products	Peanuts, peanut butter or peanut paste.
Nuts other than peanuts	
Tomato sauce or Ketchup	
Fat spreads	Cream, sour cream, or Mayonnaise.
Oil and vinegar salad dressing	
Low-calorie salad dressing	
Butter on vegetables	
Margarine or oil on vegetables	
Yeast extracts/spreads	Vegemite, Marmite, or Promite.
Jam, marmalade, honey, or syrups	
Coffee	
Tea	
Herbal tea	
Alcohol	

**Table 2:** **Food groups loading**^**a**^**>0.15 on each of the dietary patterns identified in women at baseline**

**Label of the dietary patterns**	**Food groups (loadings)**
Fruits and vegetables	Raw vegetables (0.31), legumes (0.28), cooked vegetables (0.26), non-fried fish (0.25), nuts other than peanuts (0.24), fruits (common: 0.23; others: 0.23), herbal tea (0.21), salad dressing (0.20), yogurt (0.19), olives (0.19), porridge (0.18), and other milks (such as soy milk, 0.16); and diet carbonated soft drink (-0.21).
High-energy snack and processed foods	Pizzas (0.43), savory pastries (0.42), crisps (0.32), Ketchup (0.30), peanut products (0.25), olives (0.25), yeast extracts/spreads (0.23), chocolate products (0.21), cheese (0.18), nuts other than peanuts (0.17), and jam/syrups (0.17); and rice (-0.153).
High-fat foods	Potatoes cooked with added fat (0.29), fat added to vegetables (butter on vegetables, 0.27; margarine or oil on vegetables, 0.26), white bread (0.25), fried fish (0.24), fat spreads (0.23), full cream milk (0.21), cakes and pastries (0.23), rice (0.19), sausages (0.18), sweet biscuits (0.18), non-diet carbonated soft-drink (0.16), fruit juices (0.16), red meats (0.16), chocolate-based products (0.15), and ice cream (0.15); and reduced-fat milk (-0.20).
Cereals and sweet foods	Cereals (wholemeal crackers (0.47); breakfast cereals (0.24)), confectionary other than chocolate-based (0.47), reduced-fat milk (0.28), ice cream (0.21), diet-carbonated soft drink (0.19), low-calorie salad dressing (0.16), yogurt (0.151); full cream milk (-0.26).

^a^Absolute values.

Factor loadings for food group indicated in parentheses.

The primary analytic approach was based on the intention-to-treat principle, with all participants completing follow-ups included in the condition to which they were assigned. Baseline comparability of intervention and control arms was assessed using Chi square tests (categorical variables) and linear regression analyses (continuous variables). Analysis of covariance (ANCOVA) was used to measure the impact of the intervention at post-intervention on each of the six outcomes of interest, i.e. the four dietary patterns scores, total physical activity and TV viewing time. These are regression analyses which adjust for baseline outcomes and can be summarized as follows: follow-up scores = constant + a*baseline score + b*treatment arm. The coefficient b is the effect of interest and represents the difference between the mean change scores of each treatment arm [29,30]. Resulting residuals were checked in terms of distribution and independence, and dependent variables consequently transformed when needed (square-root transformations for physical activity and TV viewing time). Additional analyses of covariance were undertaken which also accounted for classical predictors of the outcomes studied, i.e. educational level, age, and pre-pregnancy weight status defined in 2 categories (namely BMI < 25 kg/m^2^ and BMI ≥ 25 kg/m^2^). Clustering by first-time parents’ group was accounted for in all models.

The significance level was set at 5%. Analyses were conducted using Stata software (Release 10; StataCorpLP, College Station, TX, USA).

## Results

**Figure 1:**
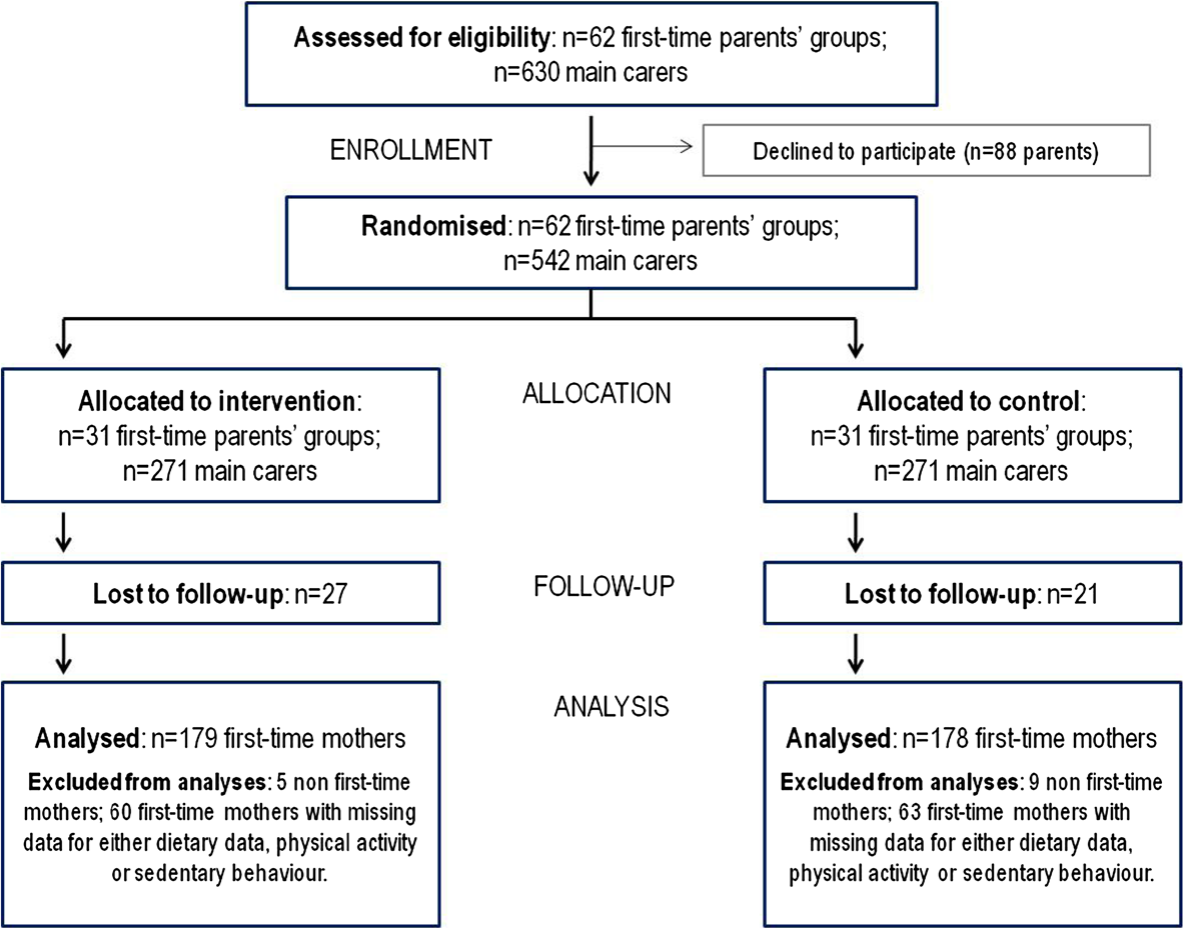
InFANT design and participant flow.

The CONSORT [30] statement flow chart (Figure1) indicates that a total of 62 first-time parent groups were enrolled in the study (84% response). Among the 630 mothers approached in these 62 groups, 88 declined to participate. Of the 542 mothers entered at baseline, 357 (66%) were available at post-intervention with complete data regarding the variables of interest for the current study. In fact, 48 mothers were lost at post-intervention; 123 had missing data either at baseline or at follow-up; and 14 mothers were excluded because they were not first-time mothers. Baseline characteristics of study participants are shown in Table3. No differences were observed at baseline in any socio-demographic variables between intervention and control groups, or between those retained or eliminated from the analyses (data not presented). 

**Table 3:** Baseline characteristics of 542 mothers and infants pairs according to treatment arm

	**All**	**Intervention**	**Control**
Sample size	542	271	271
**Children**			
Age of the new born at baseline (months)	3.9 (1.5)	3.9 (1.5)	3.8 (1.5)
Sex, % (CI95%)			
Boy	52.8 (48.5; 57.2)	52.3 (47.3; 57.3)	53.4 (46.3; 60.5)
Girl	47.2 (42.8; 51.5)	47.7 (42.7; 52.7)	46.6 (39.5; 53.7)
Currently breastfeeding the baby, % (CI95%)			
Yes^a^	71.7 (66.7; 76.7)	67.7 (60.2; 75.3)	75.7 (69.7; 81.7)
No	28.3 (23.3; 33.3)	32.3 (24.7; 39.8)	24.3 (18.3; 30.3)
**Mothers**			
Age of the mother at baseline (years)	32.3 (4.3)	32.5 (4.2)	32.0 (4.4)
BMI before pregnancy (kg/m2)	24.5 (5.3)	24.7 (5.6)	24.3 (5.0)
Education level, % (CI95%)			
Low	21.1 (17.1; 25.2)	22.0 (16.3; 27.7)	20.3 (14.6; 26.0)
Intermediate	24.7 (20.7; 28.8)	26.5 (21.0; 32.1)	22.9 (17.2; 28.6)
High	54.2 (48.1; 60.2)	51.5 (43.9; 59.1)	56.8 (47.6; 66.0)
Employment status, % (CI95%)			
On maternity leave	66.1 (62.4; 69.8)	66.8 (61.4; 72.2)	65.4 (60.4; 70.4)
Home duties full time	16.8 (13.6; 19.9)	15.6 (10.8; 20.5)	17.9 (14.0; 21.8)
Employed	9.3 (6.7; 12.0)	8.8 (5.2; 12.3)	9.9 (6.0; 13.8)
Unemployed	4.4 (2.5; 6.3)	4.2 (1.6; 6.8)	4.6 (1.7; 7.4)
Student	1.1 (0.3; 2.0)	1.9 (0.3; 3.5)	0.4 (0; 1.1)
Other	2.3 (0.8; 3.8)	2.7 (0.1; 5.2)	1.9 (0.3; 3.5)
Country of birth, % (CI95%)			
Australia	79.1 (75.0; 83.1)	78.4 (72.3; 84.5)	79.7 (74.3; 85.1)
Other	20.9 (16.9; 25.0)	21.6 (15.5; 27.7)	20.3 (14.9;25.7)
Language spoken at home, % (CI95%)			
English	93.8 (91.3; 96.2)	93.9 (89.9-98.0)	93.6 (90.8-96.4)
Other	6.2 (3.8; 8.7)	6.1 (2.0; 10.1)	6.4 (3.6; 9.2)

Figures are means (SD) unless stated otherwise.

^a^This category includes both partial and exclusive breastfeeding.

The analyses at post-intervention, both without and with adjustment for confounders [30], revealed beneficial intervention *vs.* control effects on two dietary patterns: the scores of both the "High-energy snack and processed foods" and the "High-fat foods" patterns decreased more in the intervention group (Table4), i.e. -0.22 (−0.42;-0.02), P = 0.03 and −0.25 (−0.50;-0.01), P = 0.04, indicating less adherence to undesirable dietary patterns for mothers in the intervention group at study completion. No beneficial effects were observed regarding the two other dietary patterns, total physical activity or TV viewing time. 

**Table 4:** Adjusted mothers’ dietary patterns scores, physical activity and sedentary behaviors at post-intervention

	**Baseline**				**Follow-up**							
	**Intervention**		**Control**		**Intervention**		**Control**		**Difference (intervention - control), adjusted for baseline (95% CI)**^**a**^	**P-value**	**Difference (intervention - control), adjusted for baseline and covariates (95% CI)**^**b**^	**P-value**
	n	Mean (SD)	n	Mean (SD)	n	Mean (SD)	n	Mean (SD)				
"Fruits and vegetables" pattern scores	178	0.02 (1.68)	179	-0.02 (1.53)	178	-0.06 (1.54)	179	0.06 (1.68)	-0.17 (-0.40;0.07)	0.16	-0.16 (-0.39;0.07)	0.17
"High-energy snack and processed foods" pattern scores	178	0.02 (1.25)	179	-0.02 (1.25)	178	-0.10 (1.27)	179	0.10 (1.38)	-0.22 (-0.42;-0.02)	0.03	-0.21 (-0.41;-0.02)	0.03
"High-fat foods" pattern scores	178	0.03 (1.35)	179	-0.03 (1.38)	178	-0.11 (1.26)	179	0.11 (1.57)	-0.25 (-0.50;-0.01)	0.04	-0.25 (-0.50;-0.01)	0.05
"Cereals and sweet foods" pattern scores	178	0 (1.38)	179	0 (1.24)	178	-0.09 (0.89)	179	0.09 (1.12)	-0.17 (-0.36;0.02)	0.07	-0.15 (-0.33;0.04)	0.12
Total physical activity (min/week)^c^	178	411.21 (338.62)	179	511.87 (404.47)	178	387.72 (346.28)	179	403.62 (363.79)	0.75 (-0.90;2.40)	0.37	0.84 (-0.78;2.47)	0.30
Sedentary behavior (min/day)^c^	178	219.0 (158.2)	179	203.7 (119.7)	178	162.91 (129.24)	179	150.44 (125.92)	0.22 (-0.73;1.17)	0.64	0.28 (-0.65;1.22)	0.55

^a^Adjusted for baseline value; ^b^Adjusted for baseline value, maternal education level, maternal age, and maternal pre-pregnancy weight status defined in 2 categories (namely BMI<25 kg/m^2^ and BMI≥25 kg/m^2^); ^c^Square-root transformation.

Additional moderation analyses were undertaken to assess if the effects observed differed according to subgroups, namely ‘healthy weight’ *vs.* ‘overweight (including obesity)’; ‘high education level’ *vs.* ‘intermediate or low education levels’ (results not shown). No statistical significant interaction was observed, suggesting that the effects observed in our study were not moderated either by maternal weight status or education level.

## Discussion

To our knowledge, this obesity prevention intervention is one of the first cluster-RCTs to have targeted first-time parents of infants within an existing health service infrastructure. The current study focused on lifestyle behaviors in first-time mothers post intervention and showed positive impact on two of the targeted dietary patterns, characterized mainly by high-fat or processed foods. The intervention did not influence other maternal health behaviors, having no impact on the consumption of healthier foods (as reflected in the “Fruits and vegetables” pattern), physical activity or TV viewing time.

First-time mothers represent an important target group for lifestyle interventions for several reasons. Firstly, young Australian women aged 18–30 y have gained weight faster than their older counterparts over the last decade [31]. Ball and colleagues report that this trend can be accounted for in part by a decrease in physical activity, and an increase in fast-food consumption [32]. Secondly, first-time mothers face additional barriers likely to influence the adoption and maintenance of healthy lifestyle behaviors, due to their new commitments towards child care, housework and shopping [33,34]. This is a time of transition and greater vulnerability, with significant changes in their biological life, time availability, and both domestic and social situations [32]. Given this context, first-time mothers are likely to be receptive and sensitive to advice and recommendations [35]. In fact, high levels of recruitment and retention were observed in this study (86% of all women agreed to participate, and 9% only lost to follow-up), with results further suggesting that young mothers seem responsive to changing their own behaviors. Finally, the current study provides unique evidence regarding the potential to utilize young mother’s existing social networks as a vehicle through which to deliver health promotion interventions.

The current findings give new insights into the effectiveness of multidimensional interventions, which seek to promote parenting skills for positive lifestyle behaviors; increase knowledge; provide social support; reduce perceived barriers; and encourage feelings of self-efficacy [36]. However, not all behaviors promoted through the InFANT were improved post-intervention. The absence of beneficial effects on the healthier dietary pattern (“Fruits and vegetables”) and on both physical activity and TV viewing outcomes may reflect the low “dose” of intervention regarding parenting health behaviors. Our results also suggest that young mothers may find modifying some aspects of food consumption easier to achieve than modifying their physical activity and TV behaviors. This latter hypothesis is supported by work undertaken by Ball and colleagues where young women (18–32 years) reported adoption of a range of healthy eating patterns was more achievable than the adoption of a set of physical activity behaviors [32]. In a previous RCT specifically aimed at promoting physical activity in women with young children, Miller and colleagues have demonstrated that enhancing self-efficacy and partner support through community engagement could be a means to increase physical activity in mothers [34].

Promoting active play and limiting sedentary time for children were important foci of InFANT, however more time was devoted to discussions regarding children’s food, nutrition and feeding styles. It is possible that the emphasis on child feeding strategies and promoting a healthy diet for children may have influenced mother’s own eating patterns. Indeed, an underlying premise of the design of InFANT was that parents are likely to be receptive to information which will support them to achieve their aims of providing ‘the best’ for their child [18,35]. A further explanation of our null findings for improved physical activity behaviors may relate to our measure of physical activity. In this study physical activity was assessed using a self-reported questionnaire and the precision of the resulting measurement is relatively low. It is possible that we lacked power to be able to show any significant effect of the intervention on this specific behavior. Objective measurements of physical activity, using accelerometers or pedometers for example, are recognized to provide relatively higher accuracy and precision.

With regards to the differential effect of the intervention on dietary patterns, the following hypotheses may be posited. Firstly, due to taste issues, it may be more demanding to increase consumption of healthy foods - such as those which characterize the “Fruits and vegetables” dietary patterns (i.e. raw vegetables, legumes, cooked vegetables, non-fried fish, nuts other than peanuts, and fruits) - than to reduce high-fat and processed foods. Secondly, lack of time for purchasing, storage and cooking, along with perceptions of relatively high costs of these foods, may be additional barriers for improving adherence to healthy dietary patterns during this particular stage of women’s lives [32,37]. Finally, from a methodological point of view, the FFQ did not account comprehensively for mixed recipes. As a result, we may have missed part of the vegetable consumption for example, as vegetables are often ingredients of complex dishes. We cannot exclude that mothers receiving the intervention increased their use of vegetables in mixed dishes.

### Strengths and limitations

The strengths of the current study include the cluster-RCT design and the high response and retention rates. Considering overall diet through the dietary pattern approach is an additional advantage, as compared to the traditional single-food group approach, which does not account for colinearity among all dietary components [22]. Despite subjective choices inherent to factor analysis, Newby and colleagues reported in their review that reproducibility has been observed between most studies which have identified patterns in adults [22]. This consistency over national and international studies was confirmed for the InFANT study, as previously described [26]. Some limitations of the study need to be acknowledged. First, we acknowledge that the differences between the mean change scores of each treatment arm observed for the "High-energy snack and processed foods" and the "High-fat foods" patterns are small, although statistically significant. This is likely to come from the low dose prevention intervention, as well as the challenge it is to influence maternal behaviors. Nonetheless, small changes in multiple health-related behaviors, if sustained (and thus accumulated) over the life course, are likely to favorably impact on maternal energy balance. Second, although all socio-economic positions were represented in this study, university educated women are over represented, with 54.2% reporting high education level (university degree or higher). In addition, participants in this study were older than the average age of first-time mothers in Victoria (32.3 years compared to 29.1 years) [38]. These characteristics may have implications for generalisibility. However, they may be partially explained by the inclusion of only urban Melbourne residents in the trial, who are likely to differ from the broader Victorian population. Another limitation of the study is the number of mothers excluded from the analyses at post-intervention (34%), mainly due to missing data regarding the main outcomes (123 mothers out of the total 185 excluded from the analyses). Mothers excluded from the analyses did not differ significantly from the included mothers in terms of socio-demographic characteristics. However, less statistical power was available to assess the effects of the intervention.

## Conclusion

The current findings support the hypothesis that a low intensity childhood obesity prevention intervention, which targets first-time parents in existing social groups, and focuses on parenting skills to promote positive lifestyle behaviors, may improve mothers’ dietary patterns. Further research needs to assess cost-effective ways in which we might maximize any impacts already achieved and further enhance those lifestyle behaviors not impacted by the InFANT intervention. The strengthening of the existing InFANT program with low cost, broad reach technologies is currently underway.

## Competing interests

The authors declare that they have no competing interests.

## Authors’ contributions

SL conducted the statistical analysis, contributed to interpretation of results, drafted and edited the manuscript, and had primary responsibility for final content. KJC was the principal investigator on The Melbourne InFANT Program. She designed and led that study, conducted the dietary data collection, guided the statistical analysis, contributed to interpretation of results, drafted and edited the manuscript. DC guided the statistical analysis, contributed to interpretation of results, drafted and edited the manuscript. AC S conducted the dietary data collection, drafted and edited the manuscript. KH designed and led The Melbourne InFANT Program, guided the statistical analysis, contributed to interpretation of results, drafted and edited the manuscript. SAM conducted the dietary data collection, guided the statistical analysis, contributed to interpretation of results, drafted and edited the manuscript. All authors have read and approved the final manuscript.

## Sources of support

SL is supported by a Deakin University Alfred Deakin Postdoctoral Fellowship. KJC and DC are supported by fellowships from the Victorian Health Promotion Foundation. ACS was supported by a Deakin University Postgraduate Research Scholarship. KH is supported by a National Heart Foundation of Australia Career Development Award. SAM is supported by an Australian Research Council Future Fellowship.

The Melbourne Infant Feeding Activity and Nutrition Trial (InFANT) Program was funded by an Australian National Health and Medical Research Council Project Grant (number 425801).
